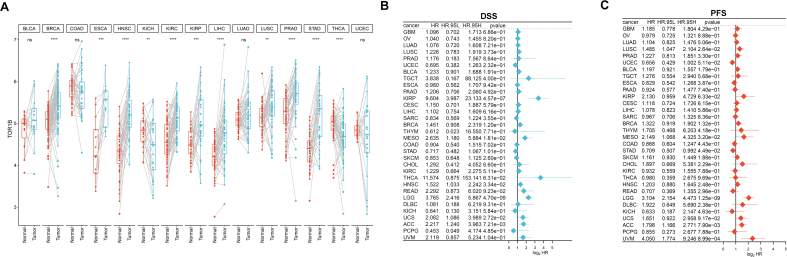# Elevated TOR1B expression predicts poor survival outcomes in patients with breast cancer

**DOI:** 10.1016/j.gendis.2025.101826

**Published:** 2025-08-21

**Authors:** Kun Fang, Suxiao Jiang, Zhengjie Xu, Meng Luo, Yinling Ma, Changsheng Yan

**Affiliations:** a Department of Surgery, Yinchuan Maternal and Child Health Hospital, Yinchuan, Ningxia 750001, China; b Department of Surgery, Yinchuan Maternal and Child Health Hospital Affiliated to Ningxia Medical University, Yinchuan, Ningxia 750001, China; c Department of Surgery, The First Affiliated Hospital of Harbin Medical University, Harbin, Heilongjiang 150001, China

Breast cancer (BRCA) is one of the most prevalent cancers globally, with high occurrence and death rates.^1^ Although the TOR1B is known to be essential for regulating cell balance and reacting to endoplasmic reticulum stress, its impact on breast cancer is not yet fully understood.^2^^,^^3^ This study is the first to provide a thorough analysis of TOR1B in BRCA. TOR1B expression was significantly elevated in tumor tissue across the GSE15852, GSE109169 and TCGA-BRCA cohorts. Patients were then divided into high and low groups according to the expression level of TOR1B. The low expression group of TOR1B was characterized by a favorable survival outcome and may benefit from immunotherapy, while the high expression group of TOR1B was associated with elevated infiltration levels of immune cells and immune checkpoints. Moreover, three drugs (ZSTK474, navitoclax, and ABT-737) from CTRP and five drugs (meclizine, NVP-BVU972, propranolol, BMS-986020, and SR-27897) from the PRISM database were screened for the high TOR1B expression group. Single-cell analysis results demonstrated that TOR1B was highly expressed in monocyte. Finally, the overexpression of TOR1B promoted the proliferation, migration, and invasion of BRCA cells. These results indicate that TOR1B is a promising prognostic marker for predicting the survival of BRCA patients.

To clarify this, we first explored the expression of TOR1B in the GSE15852, GSE109169, and TCGA-BRCA cohorts. We observed a notably increased expression level in tumor tissue compared to normal tissue across multiple cohorts (Fig. 1A and B; Fig. S1A and S1B). Immunohistochemistry, qRT-PCR and Western blot results also confirmed the expression level of TOR1B in BRCA (Fig. 1C; Figs. S2, S3A, S3B). We further categorized patients into high and low expression groups across the three cohorts based on the level of TOR1B. The Kaplan–Meier (KM) curve results identified that patients in the low expression group of TOR1B had a notably longer survival compared to those in the high expression group in terms of overall survival (OS) in the GSE17705 (Fig. S1C), GSE20685 (Fig. S1D), and TCGA-BRCA (Fig. 1D) cohorts. Similarly, we also observed significant differences in disease free survival (DFS, Fig. S1E), disease specific survival (DSS, Fig. S1F), and progression free survival (PFS, Fig. S1G) in the TCGA-BRCA cohort, which is consistent with previous results. Moreover, patients in the high expression group of TOR1B had higher mortality rates and more advanced N stages compared to those in the low expression in OS (Fig. S4A), DFS (Fig. S4B), DSS (Fig. S4C), PFS (Fig. S4D) (*P* < 0.05). We further identified 2046 differentially expressed genes between the high and low expression groups of TRO1B and then conducted Gene Ontology (GO) and Kyoto Encyclopedia of Genes and Genomes (KEGG) enrichment analyses to explore the functions or pathways involved in TRO1B (Table S1). The GO enrichment results revealed that genes were mainly involved in extracellular matrix organization, extracellular structure organization, and response to transforming growth factor beta, etc (Fig. S5A). The KEGG enrichment results showed that the genes were predominantly enriched in pathways such as the ECM-receptor interaction, focal adhesion, the TGF-beta signaling pathway, and the PI3K-Akt signaling pathway (Fig. S5B). Moreover, by performing gene set enrichment analysis (GSEA) analysis, we identified that focal adhesion, the PI3K-Akt signaling pathway, the Rap1 signaling pathway, the FOXO signaling pathway, the TGF-beta signaling and hippo signaling pathways were significantly enriched in the high expression group of TOR1B (Fig. 1E).

**Figure 1:**
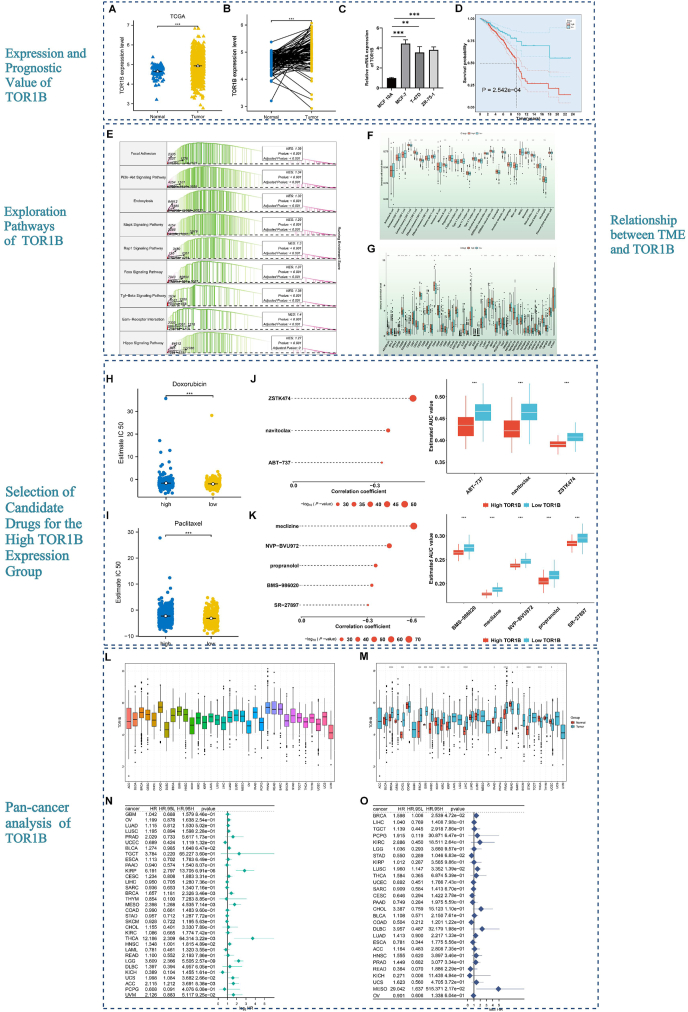
Comprehensive analysis of the role of TOR1B in BRCA. **(A)** Validation of TOR1B expression in tumor and normal tissues in the TCGA-BRCA cohort. **(B)** Evaluation of TOR1B expression in paired normal and tumor samples. **(C)** Detection of TOR1B mRNA expression in different cells via qRT-PCR. **(D)** Kaplan–Meier (KM) survival curve analysis of BRCA patients with high and low expression of TOR1B in the TCGA-BRCA cohort. **(E)** Exploration of potential pathways involved in the high expression group of TOR1B through the GSEA algorithm. **(F)** Comparisons of the infiltration of 28 immune cell types in the TOR1B high and low expression group. **(G)** Comparisons of immune checkpoints in the TOR1B high and low expression group. Drug sensitivity estimation of doxorubicin **(H)** and paclitaxel **(I)** in the TOR1B high and low expression group. Characterization of candidate agents in the CTRP **(J)** and PRISM **(K)** databases for high expression group patients. **(L)** The expression level of TOR1B in pan cancers. **(M)** Comparisons of TOR1B in normal and tumor samples in pan cancers. A forest plot was applied to estimate the prognostic value of TOR1B in TCGA-OS (**N**), TCGA-DFS (**O**). ∗*P* < 0.05, ∗∗*P* < 0.01, and ∗∗∗*P* < 0.001.

To investigate the role of TOR1B in breast cancer, we transfected a sh-TOR1B plasmid into MCF-7 cells to establish a TOR1B knockdown model. qRT-PCR and Western blot confirmed significant reductions in TOR1B mRNA and protein levels (Fig. S6A–S6C). Functional assays revealed that TOR1B knockdown significantly inhibited cell proliferation (CCK8 assay, Fig. S7A) and enhanced apoptosis, as shown by flow cytometry and TUNEL staining (Fig. S7B and S7C). Moreover, Transwell and scratch assays demonstrated reduced cell invasion and migration, respectively (Fig. S8A and S8B). These results suggest that TOR1B knockdown suppresses proliferation, promotes apoptosis, and impairs migration and invasion in breast cancer cells. Mechanistically, TOR1B knockdown reduced phosphorylated PI3K (p-PI3K) and AKT (p-AKT) levels, as shown by Western blot (Fig. S9A and S9B). Conversely, TOR1B overexpression in knockdown cells restored p-PI3K and p-AKT expression, confirming its regulatory role (Fig. S10A and S10B). A nude mouse model injected with TOR1B knockdown breast cancer cells showed slower tumor growth and smaller tumor sizes *in vivo* (Fig. S11A). Western blot analysis of tumor tissues validated the inhibition of the PI3K/AKT pathway in the knockdown group (Fig. S11B). These findings suggest that TOR1B might promote tumor progression through the PI3K/AKT pathway.

To further clarify the relationship between immune cells and TOR1B expression, we estimated 28 immune cell scores for each BRCA sample through the ssGSEA algorithm and compared them in high and low expression groups of TOR1B. As depicted in Figure 1F, most immune cells presented a higher immune infiltration level in the TOR1B high expression group compared to the low TOR1B expression group. Moreover, an increased expression level of immune checkpoints (CD274, PDCD1LG2, CD276, etc.) was observed in the high TOR1B expression group compared to the low expression group (Fig. 1G). The TIDE algorithm is utilized for evaluating tumor immune escape ability and predicting the treatment response of immune checkpoint inhibitors (ICIs). A lower TIDE value indicates that patients benefit more from immunotherapy response. Our analysis observed a significantly lower TIDE value in the low expression group of TOR1B, indicating that patients in the low expression group may benefit from immunotherapy (Fig. S12A–S12D).

To evaluate drug sensitivity between the high and low expression group of TOR1B, two drugs (doxorubicin and paclitaxel) were included in the analysis, and the results indicated that patients in the TOR1B low expression group are more sensitive to the drugs (Fig. 1H and I). We also evaluated the correlation between TOR1B and drugs, and discovered that neratinib, NMS-E628, 6-mercaptopurine, and bosutinib were highly correlated with TOR1B (Fig. S12E). Additionally, we identified three candidate drugs (ZSTK474, navitoclax, and ABT737) from CTRPP (Fig. 1J) and five candidate drugs (meclizine, NVP-BVU972, propranolol, BMS-986020, and SR-7897) from the PRISM database for patients in the high expression group of TOR1B (Fig. 1K).

We further investigated the role of TOR1B in the GSE148673 single-cell cohort. After quality control, a total of 4052 cells were identified (Fig. S13A). The correlations among the gene count, sequencing depth, and mitochondrial in the five samples were shown in Figure S13B. After selecting the top 2000 most variable genes, we utilized PCA and t-SNE algorithms for dimensionality reduction, and 26 clusters were identified (Fig. S13C). Based on the marker genes identified by distinct cells, we annotated 10 cell types, including epithelial cells, macrophages, T cells, monocytes, chondrocytes, fibroblasts, B cells, NK cells, tissue stem cells and endendrocytes (Fig. S13D and S13E). The proportion of cells was shown in Figure S13F, and we also identified that TOR1B was highly expressed in monocytes (Fig. S13G and S13H).

To explore of the role of TOR1B in pan cancers, we retrieved data on 33 cancer types from the TCGA database. As shown in Figure 1L, we observed that prostate adenocarcinoma (PARD) had the highest TOR1B level. Moreover, TOR1B was highly expressed in most tumor samples (*P* < 0.05) (Fig. 1M). The results of further paired analysis of cancers also support these findings (Fig. S14A). Univariate Cox regression analysis was performed to further confirm the prognostic value of TOR1B in OS, DFS, DSS, and PFS (Fig. 1N and O; Fig. S14B and S14C).

Overall, we thoroughly explored the role of TOR1B in BRCA. These findings offer valuable insights into the treatment and prognosis of BRCA, indicating that TOR1B is a promising biomarker for the personalized therapy and clinical management of BRCA patients.

## CRediT authorship contribution statement

**Kun Fang:** Writing – review & editing, Project administration, Funding acquisition, Conceptualization. **Suxiao Jiang:** Writing – original draft, Validation, Formal analysis. **Zhengjie Xu:** Writing – original draft, Validation, Formal analysis. **Meng Luo:** Writing – original draft, Data curation. **Yinling Ma:** Writing – original draft, Data curation. **Changsheng Yan:** Writing – original draft, Data curation.

## Funding

The research was supported by a Ningxia Reproductive Disease Clinical Medical Research Center Project, China (2023LCYX003); Ningxia Hui Autonomous Region Natural Science Foundation Project, China (2022AAC03748, 2021AAC03523); Ningxia Young Top Talent Training Program, China (NRSF-2024-106); Yinchuan Science and Technology Innovation Project, China (2023SF25); Yinchuan Academic and Technological Leader Reserve Program, China (YRCF-2024-5); Yinchuan Science and Technology Innovation Team Development Program, China (2025YCKJCX30); Basic research project of Yinchuan Maternal and Child Health Hospital, China (2024NYFYCX01, 2023NYFYCX01, 2022NYFYCX05).

## Conflict of interests

The authors declare that they have no competing interests.

## Figures and Tables

**figs1:**
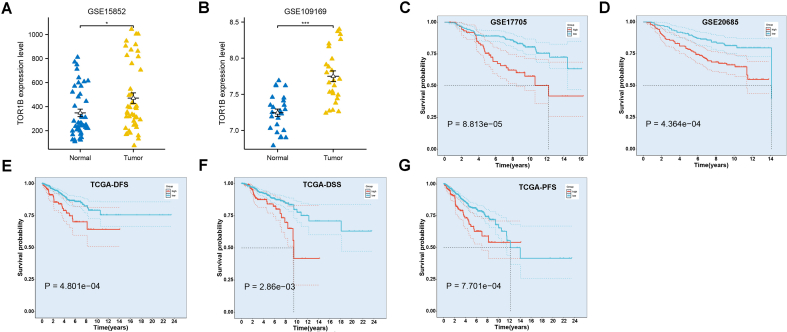


**figs2:**
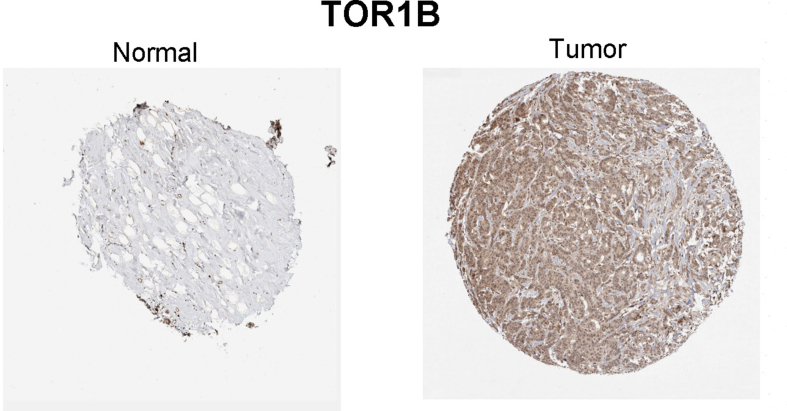


**figs3:**
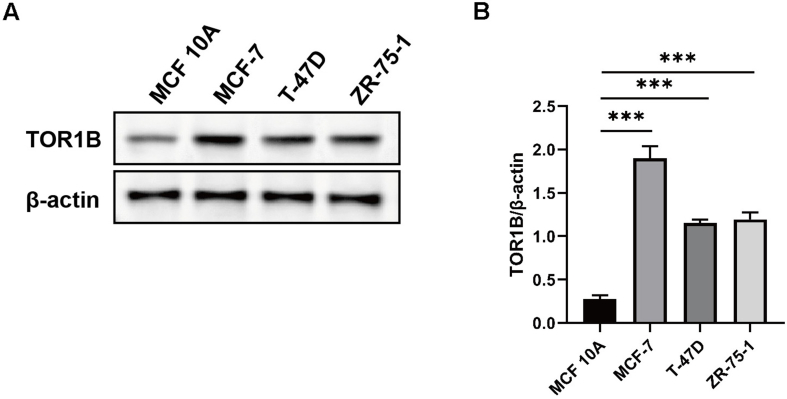


**figs4:**
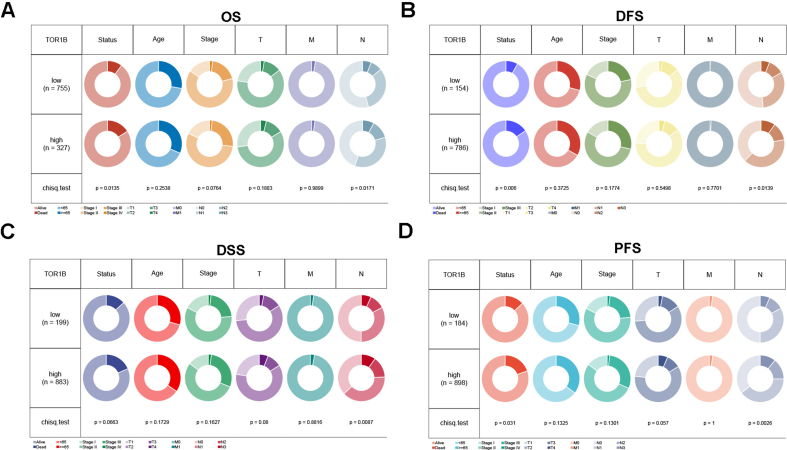


**figs5:**
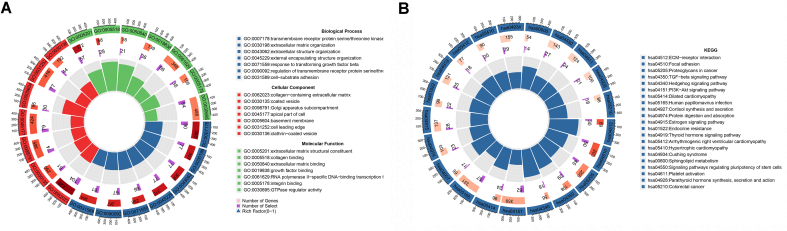


**figs6:**
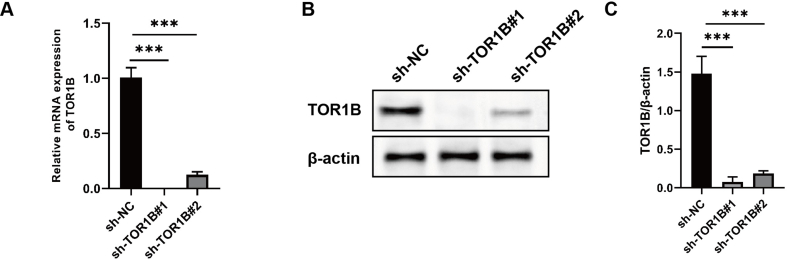


**figs7:**
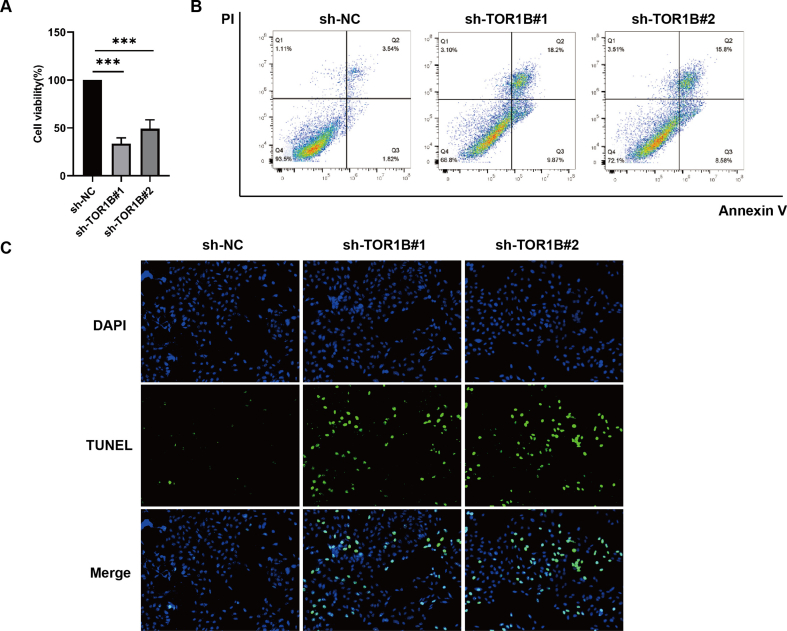


**figs8:**
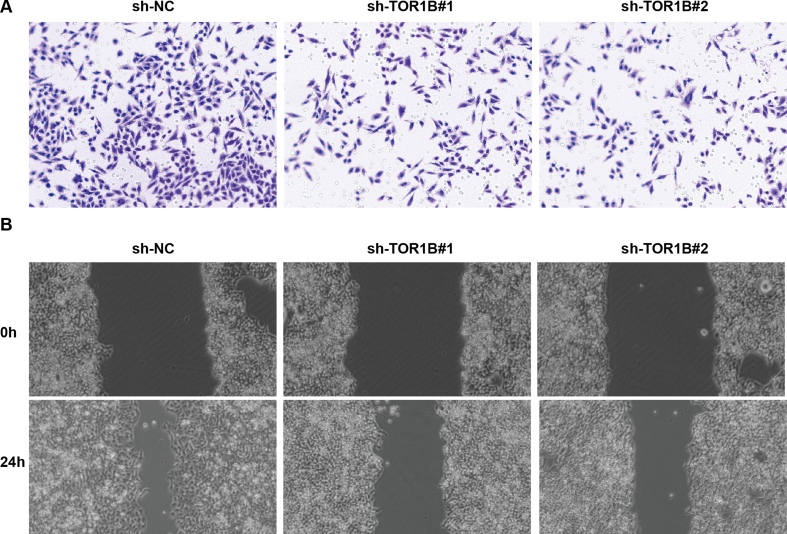


**figs9:**
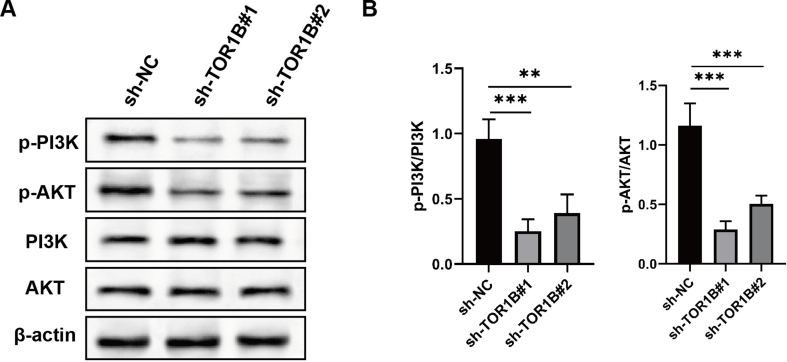


**figs10:**
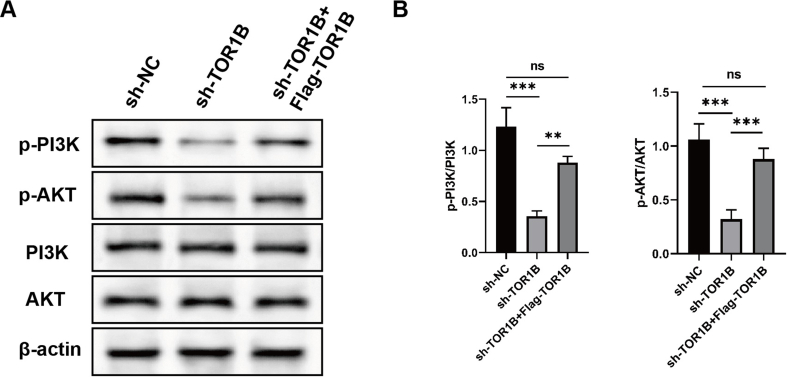


**figs11:**
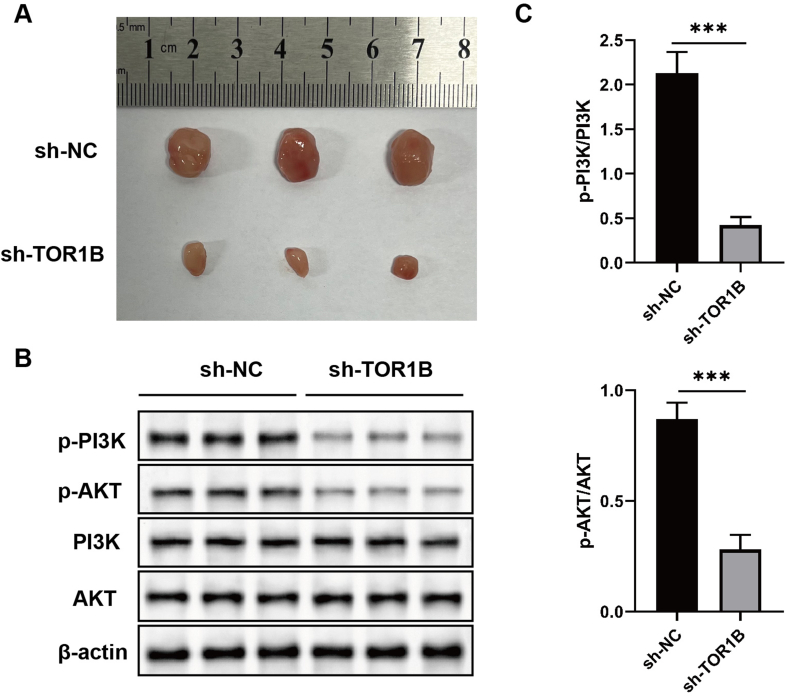


**figs12:**
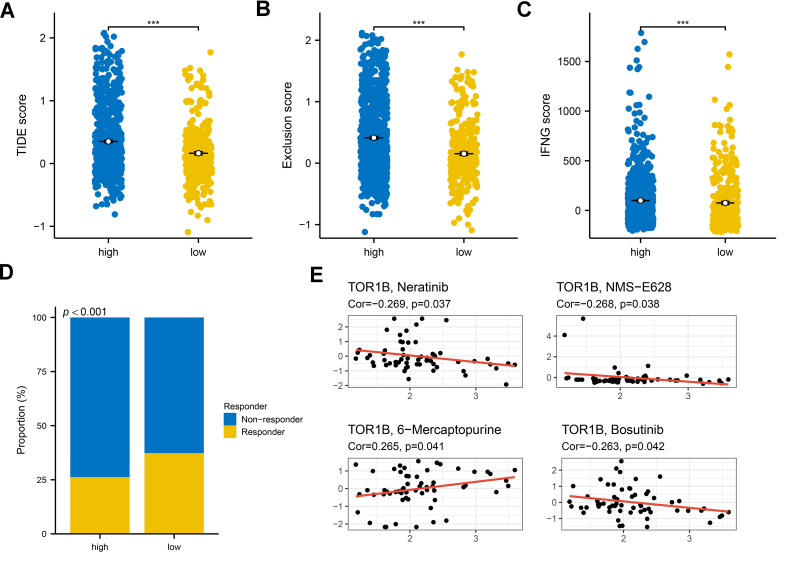


**figs13:**
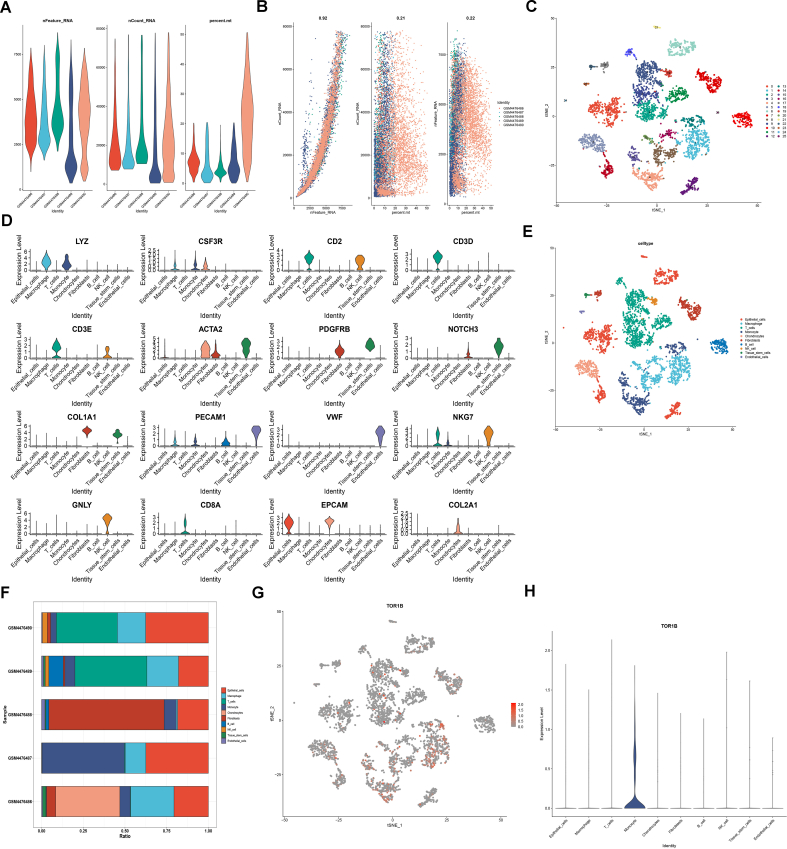


**figs14:**