# Supplementary material for: Elevated TOR1B expression predicts poor survival outcomes in patients with breast cancer
Source: Genes Dis. 2025 Aug 21;13(3):101826. doi: 10.1016/j.gendis.2025.101826 (PMC12854870; doi:10.1016/j.gendis.2025.101826)
Supplement: Multimedia component 1 [file mmc1.doc]

**Materials and Methods**

**Dataset collection and preprocessing**

Five datasets including GSE15852, GSE109169, TCGA-BRCA, GSE17705, GSE20685, along with their clinical pathological variables were retrieved from TCGA and GEO database. Among these datasets, GSE158 contained 43 normal samples and 43 tumor samples^13^, GSE109169 contained 25 normal samples and 25 tumor samples^14^, GSE17705 contained 298 tumor samples^15^, GSE20685 contained 327 tumor samples^16^, and TCGA-BRCA including 113 normal samples and 1109 tumor samples. Moreover, the single cell RNA-seq dataset was acquired from the GEO database under the accession number GSE148673. The overall design was showed in **Figure 1.**

**Function and pathway enrichment**

The gene function and pathways was explored by using the “clusterProfiler” R package^17^. The GSEA algorithm was also applied to identify potential pathways that involved in the TOR1B group (high versus low). Additionally, “GSVA” R package was applied to quantify the published signature score^18^. All significant functions or pathways were assessed based on the criterion: adjusted p value < 0.05.

**Immune cell infiltration**

Immune cell reference gene set was retrieved from previously published study^19^. To quantify the immune cell infiltration, ssGSEA was performed through “GSVA” R package.

**Single-cell RNAseq analysis**

The GSE148673 cohort contained five triple negative breast cancer (TNBC) samples. In order to ensure high confidence results, cells with nFeature_RNA ranging from 100 to 5000, ncount_RNA greater than 100, and mitochondrial gene percentage below 10% will be retained using the “Seurat” R package. Following this, a principal component analysis (PCA) was carried out on the 2000 genes that showed the most variable. The significant principal components (PCs, P<0.05) were applied to t-SNE clustering. Differentially expressed genes (DEGs) between cell clusters were screened based on the criterion: “adjusted P value < 0.05 and | log2 (fold change) | > 1”. The cells were annotated using the “SingleR” R package ^20^.

**Characterization of drugs**

The drug data of human cancer cell lines (CCLs) was retrieved from CTRP and PRISM database. CTRP database provide sensitivity data for 481 compounds across 835 CCLs, whereas the PRISM offers sensitivity data for 1448 compounds across 482 CCLs. Both the two database provide the area under the curve (AUC) values as a metric for drug sensitivity, with lower AUC values indicating high sensitivity to treatment. The missing AUC values were imputed using k-nearest neighbor (k-NN) imputation.

**Cell Culturing and Transfection**

MCF-7 cells were cultured in DMEM supplemented with 10% FBS, 100 U/mL penicillin, and 100 μg/mL streptomycin at 37°C and 5% CO2. Cells in the logarithmic growth phase were seeded into six-well plates. sh-TOR1B plasmids and negative control adenoviruses were transfected according to the manufacturer's instructions. Groups included sh-NC, sh-TOR1B#1, and sh-TOR1B#2.

**CCK-8 detects cell proliferation**

Cells were seeded in 96-well plates (100 μL, 3×10^4 cells/mL). After 24 hours, 10–20 μL of CCK-8 reagent was added per well, incubated for 2 hours, and absorbance was measured at 450 nm.

**qRT-PCR experiment**

Total RNA was extracted using Trizol, and cDNA was synthesized. qRT-PCR was performed with GAPDH as an internal control. Amplification conditions included 40 cycles of 95°C for 30 s and 60°C for 30 s. Primer sequences are detailed in Table S1.

**T**able S1 Primer sequences

| Genes | Forward Primer | Reverse Primer |
| --- | --- | --- |
| β-actin | 5'-CTCCATCCTGGCCTCGCTGT-3' | 5'-GCTGTCACCTTCACCGTTCC-3' |
| TOR1B | 5'-TCAATAATAAACACAGTGGCCTG-3' | 5'-AACAGGCAAAACCCCAAAGC-3' |

**Western blot**

Proteins were extracted, separated by SDS-PAGE, and transferred to PVDF membranes. Membranes were blocked, incubated with primary (1:1000) and secondary antibodies (1:5000), and visualized using a gel imaging system.

**Cell Scratching Experiment**

Cells at 70% confluency in six-well plates were scratched with sterile tips, washed with PBS, and incubated with fresh medium. Images were taken at 0 and 24 hours.

**Transwell**

Transwell chambers with 8 μm pores were coated with Matrigel. Cells (3×10^5/mL) were seeded in serum-free DMEM in the upper chamber, with complete medium in the lower chamber. After 24 hours, cells were fixed, stained with crystal violet, and counted under a microscope.

**TUNEL staining**

Cells were fixed with 4% paraformaldehyde, permeabilized, and stained using the TUNEL assay. Fluorescence images were captured using a microscope with appropriate filters.

**Flow assay for apoptosis**

Apoptosis was detected using the Membrane-Associated Protein V-FITC/PI Apoptosis Detection Kit (ThermoFisher Scientific, USA). Cells in each group were rinsed with pre-cooled PBS, collected and diluted in 500 μL of 1× binding buffer to a concentration of 5×10^5^ cells. Then, 5 μL of Annexin V-FITC and 10 μL of PI were added to each tube. the cells were gently vortexed and incubated for 5 minutes at room temperature in the dark. The fluorescence signals of Annexin V-FITC (Ex=488 nm; Em=530 nm) and PI (Ex=535 nm; Em=615 nm) were detected by flow cytometry. The results were analyzed using FlowJo software.

**Tumor formation in nude mice**

Twelve four-week-old male Balb/C nude mice were reared and observed for one week in an SPF environment. The mice were randomly divided into 2 groups of 6 mice each according to the experimental requirements. Breast cancer cells transfected with the target plasmid DNA were cultured and collected. The injection volume of each nude mouse was kept consistent, and the cells were resuspended in complete media and transported to the animal house in an ice box. 100 μL of complete medium containing 4×10^6^ cells was injected subcutaneously into the dorsum of nude mice using a 1 mL syringe. The weight of the mice and the long and short diameters of the tumors were recorded every 3 days until the maximum length of the tumor did not exceed 20 mm. On the 15th day, the nude mice were euthanized by cervical dislocation, the tumor tissues were peeled off, photographed and recorded, and subsequent experiments were performed.

**Statistically analysis**

The R software version 4.2.2 was used for all statistical analyses. The association between TOR1B and clinical pathological variables was assessed using either the chi-square test test. Additionally, a Student’s t-test or Wilcoxon rank sum test was conducted to compare the high and low expression groups of TOR1B. The Pearson or Spearman coefficient was serve as a measure to evaluate correlation. Patients were separated into high and low TOR1B expression group on the basis of the optimal cutoff calculated by the “survminer” R package in all cohorts. ROC analysis was conducted to estimate prognostic ability of TOR1B. KM survival curve was applied to evaluate survival difference between high and low TOR1B level. In all analyses, statistical significance was determined by a p-value of less than 0.05.

**Supplementary files**

**Figure S1** Estimation of the expression and prognostic value of TOR1B in BRCA cohorts. Validation of TOR1B expression in tumor and normal tissues in GSE15852 **(A)** and GSE109169 **(B)**. Kaplan-Meier (KM) survival curve analysis of BRCA patients with high and low expression of TOR1B in the GSE17705 **(C)**, GSE20685 **(D)**, TCGA-DFS **(E),** TCGA-DSS **(F)** and TCGA-PFS **(G)** cohorts, respectively. **P* < 0.05, ***P* < 0.01, and ****P* < 0.001.

**Figure S2** Estimation of the protein level of TOR1B in the HPA database.

**Figure S3** TOR1B expression in breast cancer. **(A)** Western blot analysis of TOR1B protein expression in different cells; **(B)** Quantitative analysis of protein expression, **P* < 0.05, ***P* < 0.01, and ****P* < 0.001.

**Figure S4** The relationship between clinical pathological variables and TOR1B in the TCGA-OS **(A)**, TCGA-DFS **(B)**, TCGA-DSS **(C)** and TCGA-PFS **(D)** cohorts.

**Figure S5** Function and pathway exploration of TOR1B in the BRCA cohort. **(A)** GO and **(B)** KEGG pathway enrichment analysis of TOR1B-associated differentially expressed genes.

**Figure S6** Constructing MCF-7 cell lines knocked down for TOR1B. **(A)** Detection of TOR1B mRNA expression in different cells via qRT-PCR. **(B)** Western blot analysis of TOR1B protein expression in different cells. **(C)** Quantitative analysis of protein expression, **P* < 0.05, ***P* < 0.01, and ****P* < 0.001.

**Figure S7** sh-TOR1B inhibits breast cancer cell proliferation and promotes apoptosis. **(A)** CCK8 was used to detect the proliferative capacity of cells in different groups. **(B)** Flow cytometry was performed to detect the apoptotic capacity of the different groups. **(C)** TUNEL staining was performed to detect the apoptotic ability of the different groups, **P* < 0.05, ***P* < 0.01, and ****P* < 0.001.

**Figure S8** sh-TOR1B inhibits breast cancer cell migration and invasion. **(A)** Transwell assay for cell invasion ability in different groups. **(B)** A scratch assay was used to detect the cell migration ability of the different groups.

**Figure S9** sh-TOR1B inhibits the PI3K/AKT signaling pathway. **(A)** Western blot was performed to detect the expression of different proteins in different groups. **(B)** Quantitative analysis of protein expression, **P* < 0.05, ***P* < 0.01, and ****P* < 0.001.

**Figure S10** Overexpression of TOR1B reverses sh-TOR1B inhibition of the PI3K/AKT signaling pathway. **(A)** Western blot was performed to detect the expression of different proteins in different groups. **(B)** Quantitative analysis of protein expression, **P* < 0.05, ***P* < 0.01, and ****P* < 0.001.

**Figure S11** sh-TOR1B inhibits tumor proliferation by regulating the PI3K/AKT signaling pathway. **(A)** Tumor size in different groups of nude mice. **(B)** Western blot was performed to detect the expression of different proteins in different groups. **(C)** Quantitative analysis of protein expression, **P* < 0.05, ***P* < 0.01, and ****P* < 0.001.

**Figure S12** Immunotherapy response and drug identification of TORB1. Comparison of TIDE value **(A)**, exclusion score **(B)** and IFNG value **(C)** in the high and low TOR1B expression group. **(D)** Evaluation of the immunotherapy response in the high and low expression group. **(E)** Correlation analysis between chemotherapy drugs and TOR1B expression through cell line analysis. **P* < 0.05, ***P* < 0.01, and ****P* < 0.001.

**Figure S13** Evaluation of TOR1B at the single cell level. **(A, B)** Quality control and correlation of gene count, sequencing depth, and mitochondrial proportion in five samples from the GSE148673 cohort. **(C)** The single cells were categorized into distinct clusters through t-SNE dimension reduction. **(D, E)** Characterization of different cell types based on cell marker genes. **(F)** The proportion of different cell types in five samples. **(G, H)** Evaluation of TOR1B expression in distinct cell types.

**Figure S14** The expression and prognostic value of TOR1B in pan cancers. **(A)** The comparisons of TOR1B in paired normal and tumor samples in pan cancers. A forest plot was applied to estimate the prognostic value of TOR1B in the TCGA-DSS **(B)** and TCGA-PFS **(C)** cohorts, **P* < 0.05, ***P* < 0.01, and ****P* < 0.001.
